# Recalculation of the late fetal death rate in Mexico, 2013–2023 using ICD-11 criteria applied to national vital statistics

**DOI:** 10.1186/s40748-026-00284-5

**Published:** 2026-08-10

**Authors:** Elia Lara Lona, Pierre Buekens, Joel Martínez Soto, Juana Rosalba García Ramírez, Gerardo Chávez Saavedra, Yessica Ivet Cienfuegos Martínez

**Affiliations:** 1 Department of Medicine, University of Guanajuato, León, Guanajuato Mexico; 2 Celia Scott Weatherhead School of Public Health and Tropical Medicine, Tulane University, New Orleans, LA USA; 3 Department of Psychology, University of Guanajuato, León, Guanajuato Mexico

**Keywords:** MeSH: Fetal death, Fetal, Gestational age, Birthweight, Vital statistics, International classification of diseases, Mexico

## Abstract

**Background:**

Cross-national comparability of fetal mortality indicators is limited by heterogeneous definitions and denominators. Mexico’s official fetal death indicators include all gestational ages and use live births as the denominator, diverging from international standards. We recalculated late fetal death rates using ICD-11 criteria and compared them with published national and international data.

**Methods:**

We conducted a retrospective analysis of national fetal death data from Mexico (2013-2023). Publicly available statistics on fetal deaths and registered live-birth datasets were used. All procedures followed ICD-11 recommendations for international reporting standards. Recalculated rates were compared with official Mexican publications, PAHO/WHO estimates for Mexico, and United States (US) rates.

**Results:**

From 2013 to 2023, 254,567 fetal deaths were recorded in Mexico. The recalculated ICD-11 late fetal death rate averaged 4.9 per 1,000 total births, increasing from 4.5 (2013-2015) to 5.5 (2020-2023). However, this increase should be interpreted with caution, as it is largely driven by a decline in the number of registered live births over the study period. Official Mexican fetal death rates ranged from 10.1 to 15.5 per 1,000. PAHO/WHO estimates for Mexico (2013-2023) were 7.0-7.9 per 1,000, and US rates (2014-2023) were 2.7-2.9 per 1,000. Most fetal deaths occurred before 28 weeks, with increasing proportions among cases ≤499 g. Most deaths were classified as antepartum (84%).

**Conclusions:**

The recalculated ICD-11 late fetal death rate increased from 2013 to 2023. While national late fetal death rates were lower than historical official rates, they were nearly twice as high as US rates.

**Trial registration:**

Not applicable.

## Background

The registration of births and deaths is a core public health function that supports population health assessment, international monitoring, and evidence‑based policy development [1–3]. Despite improvements in vital statistics systems, fetal deaths (FDs) remain difficult to measure accurately across settings, particularly in low‑ and middle‑income countries [4–7]. Challenges include variability in gestational age assessment, cause‑of‑death attribution, and legal or administrative frameworks governing the recognition and registration of pregnancy [2].

Substantial heterogeneity exists in how countries define and report FDs, often deviating from recommendations of the International Classification of Diseases (ICD) [8–11]. Under ICD-10, countries were allowed considerable flexibility in defining operational thresholds for FDs, resulting in indicators that are often not comparable across time or between countries [12]. ICD-10 recommended that published rates and ratios could use a denominator of either live births or total births (live births and FDs); additionally, it advised that fetuses weighing less than 500 g or less than 22 completed weeks of gestation should be excluded from the numerator [12]. Conversely, under ICD-11, the late fetal death rate is defined as the number of fetal deaths occurring at ≥ 28 completed weeks of gestation divided by total births (live births plus fetal deaths), multiplied by 1,000 [13]. In this study, the term “fetal death” is used as a general descriptor of all fetal deaths, while “late fetal death” refers specifically to deaths occurring at ≥ 28 completed weeks of gestation, consistent with ICD-11 criteria. The term “stillbirth” is not used to avoid ambiguity and ensure consistent terminology throughout the manuscript.

The Every Newborn Action Plan (ENAP) implements the Global Strategy for Women’s and Children’s Health and aims to end preventable FDs ≥ 28 weeks, as well as to set the following goals: (1) all countries will reach the target late FD rate of 12 or less fetal deaths by 2030; and (2) all countries will reach the target late FD rate of 10 or less per 1000 total births by 2035.

Mexico maintains a consolidated administrative system for registering FD rates since 1984 [14]. In Mexico, official fetal death statistics include deaths from ≥ 12 weeks of gestation and are reported using different denominators and data capture approaches. For example, rates may be expressed per 1,000 live births or per women of reproductive age, depending on the reporting framework. Fetal deaths are reported across the full spectrum of gestational ages, including very early (12–19 weeks), early (20–27 weeks), and late (≥ 28 weeks), indicating that the official system includes all fetal deaths from ≥ 12 weeks of gestation [15–16]. These data are generated through multiple components of the Mexican National System of Statistical and Geographic Information (SNIEG), including civil registration systems and health information systems [15–16].

These definitions differ substantially from international standards, which define late fetal death using thresholds such as ≥ 28 weeks of gestation or ≥ 1,000 g birthweight and use total births (live births plus fetal deaths) as the denominator [12, 13].

Given Mexico’s pronounced regional inequalities in access to maternal care [17], the use of non-standardized criteria may obscure preventable fetal mortality patterns and hinder monitoring of progress toward global reproductive health targets.

Mexico’s size, demographic diversity, and regional location in North America further underscore the importance of harmonized FD measurement. Improving the measurement of FDs in Mexico using ICD-11 provides an opportunity to strengthen cross-national comparability with high-income countries such as the US, where standardized fetal mortality indicators are well established and where there is a large population of Mexican origin. Given the deep demographic, socioeconomic, and health-system linkages between both countries, harmonized metrics would allow more accurate regional surveillance, support binational public health initiatives, and contribute to understanding preventable perinatal mortality across different healthcare settings. By generating comparable and methodologically robust data, this study enhances the evidence base needed to inform global discussions on reproductive health measurement and equity.

To address these gaps, this study aims to: (1) recalculate late FD rates in Mexico from 2013 to 2023 using ICD-11 criteria; (2) compare recalculated rates with those published in national and international sources; and (3) describe the epidemiological distribution of FD by gestational age, birthweight, timing of death, and skin condition.

## Methods

We conducted an observational, retrospective secondary analysis of FDs occurring in Mexico between 2013 and 2023. The study followed the STROBE Statement for cross-sectional studies [18] and used publicly available national administrative data [16].

### Study design and data sources

FD records were obtained from the official Statistics on Fetal Deaths, published by the National Institute of Statistics and Geography (INEGI) [16, 19]. These datasets compile all FDs registered in Mexico. Information is derived from FD certificates collected through civil registry offices; since 2022, records have also been compiled via Secretary of Health reporting systems. Certificate models used during the study period correspond to the 2012 [20], 2017 [21], and 2022 [22] versions, and live birth data for 2013–2020 were obtained from INEGI birth statistics, while data for 2021–2023 were derived from the Secretary of Health (DGIS), reflecting the increasing use of health information system data (SINAC) in recent years. Both sources are part of the Mexican National System of Statistical and Geographic Information (SNIEG), and the System of Information on Births has been officially designated as Information of National Interest. Although differences in data collection processes may affect strict comparability, both sources are based on national administrative records of registered live births [23, 24].

For comparison purposes, we extracted late FD rates indicators from the Pan American Health Organization (PAHO), the World Health Organization (WHO) [25], and the US Centers for Disease Control and Prevention (CDC) [26]. PAHO/WHO estimates are based on a combined criterion (≥ 1,000 g or ≥ 28 weeks) and standardized definitions for international comparability.

### Case definitions and inclusion criteria

ICD-11 criteria were applied to identify late fetal deaths, defined as fetal deaths occurring at ≥ 28 completed weeks of gestation, consistent with international reporting standards. The numerator included only cases with reported gestational age meeting this threshold. Although ICD-11 permits the use of birthweight criteria (e.g., ≥ 1,000 g) when gestational age is unavailable, gestational age was used as the primary classification variable to ensure consistency and transparency in the analysis. This approach aligns with ICD-11 principles emphasizing standardized definitions to support comparability across populations and over time [13]. All FDs registered in the databases were included, irrespective of place of occurrence or type of facility. All cases meeting ICD-11 criteria were retained. Artificial termination of pregnancy is not identified in the dataset and therefore could not be excluded analytically [19].

### Variables

Available variables included:

- Gestational age, recorded in completed weeks (12^+ 0^ and 42^+ 6^ weeks) categorized as very early (12^+ 0^ to 21^+ 6^), early (22^+ 0^ to 27^+ 6^), late (28^+ 0^ to 42^+ 6^) or not specified (NS);
- Birthweight in grams (001 to 5,000), grouped as ≤ 499 g, 500–999 g, 1,000–5,000 g, or not specified (NS);
- Timing of death, classified as antepartum, intrapartum, or not specified (NS);
- Skin condition at birth grouped as fresh, macerated, or not specified (NS).

These variables allowed stratification aligned with international comparability standards.

### Statistical analysis

All datasets were downloaded in .csv format and imported into IBM SPSS Statistics v25.0 for cleaning and analysis. Descriptive statistics were computed for absolute and relative annual frequencies. ICD-11 criteria were applied to define late fetal deaths as those occurring at ≥ 28 completed weeks of gestation. The rate was calculated using total births (live births plus fetal deaths) as the denominator, in accordance with international standards and multiplied by 1,000. This definition differs from official Mexican fetal death statistics, which include fetal deaths from ≥ 12 weeks of gestation and commonly use live births as the denominator. The use of standardized ICD-11 criteria in this study was intended to improve comparability with international estimates. No reclassification based on birthweight was performed, and gestational age was used as the primary criterion.

Percent change was calculated using 2013 as the reference year. Recalculated rates were compared with those published by INEGI [16], PAHO/WHO (Mexico) [25], and US CDC Vital Statistics [26]. Cross-tabulations were generated to examine distributions by gestational age, birthweight, timing of death, and skin condition. Annual Excel files were created to document recalculated values and comparison indicators. All procedures followed ICD-11 recommendations for international reporting standards [13].

To assess temporal trends in the distribution of fetal deaths across gestational-age categories over time, we used the extended Mantel–Haenszel chi-square test for linear trend, which evaluates changes in the proportion of cases across ordered categories [27].

## Results

A total of 254,567 FDs were recorded in Mexico between 2013 and 2023, with an annual average of 23,142 FDs and 9302 late FDs (Table 1). Using ICD-11 criteria,

The recalculated late fetal death rate averaged 4.9 per 1,000 total births and increased to about 5.5 per 1,000 during 2020–2023. However, this increase should be interpreted with caution. The absolute number of late fetal deaths decreased over the study period, while the number of registered live births declined substantially.

Official INEGI FD rates, which include all gestational ages and use only live births as the denominator, ranged from 10.1 to 15.5 per 1,000 during 2013–2023, which is approximately two to three times higher than the recalculated ICD-11 late FD rate (Table 1).

**Fig. 1:**
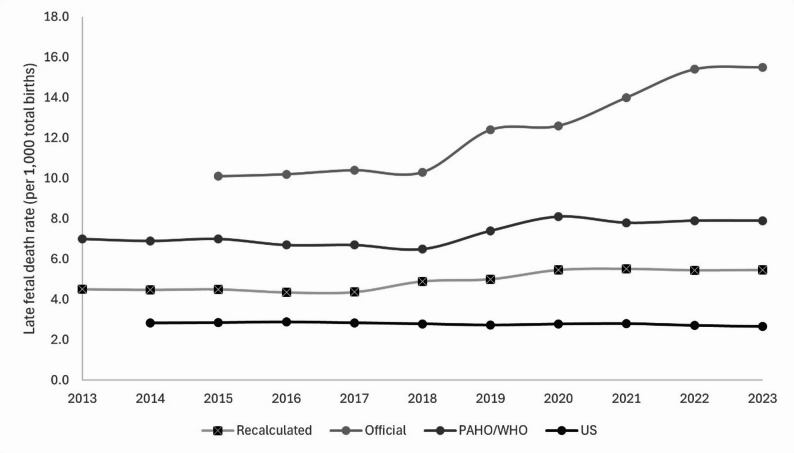
Trends in late fetal death rates in Mexico, 2013–2023. Late fetal death rates are expressed per 1,000 total births (live births plus fetal deaths). Recalculated rates are based on ICD-11 criteria (≥ 28 completed weeks). PAHO/WHO estimates use standardized definitions (≥ 1,000 g or ≥ 28 weeks) and total births. US rates correspond to ≥ 28 weeks using national vital statistics. Official Mexican estimates include fetal deaths from ≥ 12 weeks of gestation and are commonly calculated using live births as the denominator; therefore, they are not directly comparable and are shown for reference. Data are derived from Table 1

PAHO/WHO late FD rates for Mexico ranged from 7.0 to 7.9 per 1,000 births (average: 7.3). US CDC late FDs rates ranged from 2.7 to 2.9 per 1,000 births between 2014 and 2023 (Table 1). These trends are illustrated in Fig. 1.

**Table 1:** Recalculated late fetal death rate in Mexico (2013–2023) and comparison with national and international estimates

Year of occurrence	A. Total fetal deaths ^1^	B. Late Fetal Death ^2^	C. Registered live births ^3^	D. Total births (late fetal deaths + live births) ^4^	E. Recalculated Late fetal death rate for Mexico ^5^	F. Official Mexican estimates ^6^	G. PAHO/WHO estimates ^7^	H. U.S. late fetal death rate ^8^
	*N*	*n*	*N*					
2013	22,578	9921	2,190,258	2,200,179	4.5	ND	7.0	ND
2014	22,511	9763	2,171,869	2,181,632	4.5	ND	6.9	2.8
2015	22,703	9700	2,145,199	2,154,899	4.5	10.1	7.0	2.9
2016	22,208	9090	2,080,253	2,089,343	4.4	10.2	6.7	2.9
2017	22,336	9028	2,059,158	2,068,186	4.4	10.4	6.7	2.8
2018	24,144	9542	1,940,656	1,950,198	4.9	10.3	6.5	2.8
2019	23,868	9387	1,868,214	1,877,601	5.0	12.4	7.4	2.7
2020	22,637	9588	1,747,847	1,757,435	5.5	12.6	8.1	2.8
2021	23,000	9084	1,639,479	1,648,563	5.5	14.0	7.8	2.8
2022	25,041	8875	1,622,921	1,631,796	5.4	15.4	7.9	2.7
2023	23,541	8341	1,521,280	1,529,621	5.5	15.5	7.9	2.7
Total	*254,567*	*102,319*	*20,987,134*	*21,089,453*				
Average	23,142	9302	1,907,921	1,917,223	4.9	12.3	7.3	2.8

^1^ Total fetal deaths of any gestational age, from INEGI Fetal Death Statistics (EDF)

^2^ Late fetal deaths defined as ≥ 28 weeks of gestation according to ICD11

^3^ Registered live births obtained from INEGI birth statistics (2013–2020) and from the Secretary of Health (DGIS) for 2021–2023

^4^ Total births = late fetal deaths + live births

^5^ Late fetal death rate recalculated as: (late fetal deaths ≥ 28 weeks / total births) × 1000

^6^ Official Mexican fetal death statistics (INEGI): include fetal deaths from ≥ 12 weeks of gestation by 1,000 live births

^7^ PAHO/WHO estimates use standardized definitions (≥ 1,000 g or ≥ 28 weeks) and total births as the denominator

^8^ U.S. rates obtained from CDC/NCHS definitions of late fetal death ≥ 28 weeks

We also examined the distribution by gestational age and birthweight for all FDs. In 2013, 22,578 FDs were registered, compared with 23,541 in 2023, an absolute increase of 963 cases (Table 2). However, the highest numbers of FD were observed in 2018 and 2022. When considering a 10-year comparison, the absolute increase is substantially larger: 2,463 cases between 2013 and 2022, which is more than twice the increase observed between 2013 and 2023. The difference between 2013 and 2018 is 1,566 cases.

**Table 2:** Distribution of fetal deaths by gestational age and birthweight groups, Mexico, 2013–2023

Year of occurrence	Total fetal deaths	Weeks of gestation group				Birthweight group (g)			
		12–21	22–27	≥ 28	NS	≤ 499	500–999	≥ 1000	NS
	*N*	*n* (%)				*n* (%)			
2013	22,578	6966 (30.9)	5581 (24.7)	9921 (43.9)	110 (0.5)	7411 (32.8)	4546 (20.1)	10,591 (46.9)	30 (0.1)
2014	22,511	7290 (32.4)	5348 (23.8)	9763 (43.4)	110 (0.5)	7813 (34.7)	4260 (18.9)	10,396 (46.2)	42 (0.2)
2015	22,703	7516 (33.1)	5482 (24.1)	9700 (42.7)	5 (0.0)	7836 (34.5)	4342 (19.1)	10,221 (45.0)	304 (1.3)
2016	22,208	7739 (34.8)	5368 (24.2)	9090 (40.9)	11 (0.0)	8071 (36.3)	4254 (19.2)	9587 (43.2)	296 (1.3)
2017	22,336	7757 (34.7)	5539 (24.8)	9028 (40.4)	12 (0.1)	8136 (36.4)	4379 (19.6)	9495 (42.5)	326 (1.5)
2018	24,144	8666 (35.9)	5927 (24.5)	9542 (39.5)	9 (0.0)	9110 (37.7)	4646 (19.2)	10,033 (41.6)	355 (1.5)
2019	23,868	8657 (36.3)	5807 (24.3)	9387 (39.3)	17 (0.1)	9216 (38.6)	4612 (19.3)	9968 (41.8)	72 (0.3)
2020	22,637	7577 (33.5)	5471 (24.2)	9588 (42.4)	1 (0.0)	8047 (35.5)	4333 (19.1)	10,193 (45.0)	64 (0.3)
2021	23,000	8246 (35.9)	5594 (24.3)	9084 (39.5)	76 (0.3)	8860 (38.5)	4406 (19.2)	9658 (42.0)	76 (0.3)
2022	25,041	9984 (39.9)	6113 (24.4)	8875 (35.4)	69 (0.3)	10,621 (42.4)	4862 (19.4)	9486 (37.9)	72 (0.3)
2023	23,541	9236 (39.2)	5881(25.0)	8341 (35.4)	83 (0.4)	9942 (42.2)	4664 (19.8)	8896 (37.8)	39 (0.2)
Total	254,567	89,634 (35.2)	62,111 (24.4)	102,319 (40.2)	503 (0.2)	95,063 (37.3)	49,304 (19.4)	108,524 (42.6)	1676 (0.7)
Mean	23,142	8149 (35.1)	5646 (24.4)	9302 (40.3)	46 (0.2)	8642 (37.3)	4482 (19.4)	9865.8 (42.7)	152 (0.7)

Values are presented as n (%)

Gestational age categories are defined as 12–21 weeks, 22–27 weeks, and ≥28 weeks of gestation

Birthweight categories are defined as ≤499 g, 500–999 g, and ≥1000 g

NS: not specified

The proportion of late fetal deaths (≥ 28 weeks) among all fetal deaths decreased over the study period. In contrast, the proportion of early fetal deaths (22^+ 0^ to 27^+ 6^ weeks) increased slightly from 24.7% in 2013 to 25.0% in 2023, while the proportion of very early fetal deaths (12^+ 0^ to 21^+ 6^ weeks) increased more markedly from 30.9% to 39.2%. Together, these changes indicate a shift in the distribution of fetal deaths toward earlier gestational ages.

This pattern is supported by the analysis of temporal trends. Specifically, the proportion of very early fetal deaths showed a statistically significant increasing trend over time (Mantel–Haenszel chi-square test for trend, *p* < 0.001), whereas early fetal deaths showed no statistically significant trend (*p* = 0.18). In contrast, the proportion of late fetal deaths showed a statistically significant decreasing trend over the study period (*p* < 0.001).

The proportion of FDs with birthweight ≤ 499 g increased from 32.8% to 42.2%, whereas the proportion with birthweight 1,000 to 5,000 g decreased from 46.9% to 37.8% during the same period.

Cross-tabulation of gestational age and birthweight (Table 3) showed that 37.3% of all FDs weighed ≤ 499 g, 19.4% weighed 500–999 g, and 42.6% weighed 1,000–5,000 g. Among very early FDs, 99% weighed ≤ 499 g; among early FDs, 78.0% weighed 500–999 g; and among late FDs, 98.8% weighed 1,000–5,000 g.

**Table 3:** Gestational age groups by birthweight category among fetal deaths, Mexico 2013–2023

Weeks of gestation group	Birthweight group (g)				
	≤ 499	500–999	≥ 1000	NS	N
	n (%)				
12–21	88,764 (99.0)	0 (0.0)	0 (0.0)	870 (1.0)	89,634
22–27	6159 (9.9)	48,427 (78.0)	7282 (11.7)	243 (0.4)	62,111
≥ 28	1 (0.0)	798 (0.8)	101,078 (98.8)	442 (0.4)	102,319
NS	139 (27.6)	79 (15.7)	164 (32.6)	121 (24.1)	503

Values are presented as n (%)

Gestational age categories are defined as 12–21 weeks, 22–27 weeks, and ≥28 weeks of gestation

Birthweight categories are defined as ≤499 g, 500–999 g, and ≥1000 g

NS: not specified

Overall, 84.2% of FDs were classified as antepartum (Table 4). Intrapartum deaths were more frequent in the early FDs (22^+ 0^ to 27^+ 6^ weeks) at 25.3%. Fresh skin appearance predominated at earlier gestational ages, while maceration increased with advancing gestation, reaching 54.9% in the ≥ 28 weeks. Skin condition was not specified in 4.8% of cases.

**Table 4:** Timing of fetal death and condition at birth by gestational age group, Mexico 2013–2023

Weeks of gestation group	Antepartum	Intrapartum	NS	Total	Fresh (normal)	Macerated	NS
	*n* %			*N*	*n *%		
12–21	76,065 (84.9)	11,580 (12.9)	1989 (2.2)	89,634	61,962 (69.1)	22,888 (25.5)	4784 (5.3)
22–27	45,361 (73.0)	15,704 (25.3)	1046 (1.7)	62,111	40,411 (65.1)	19,006 (30.6)	2694 (4.3)
≥ 28	92,481 (90.4)	8574 (8.4)	1264 (1.2)	102,319	41,567 (40.6)	56,142 (54.9)	4610 (4.5)
NS	436 (86.7)	30 (6.0)	37 (7.4)	503	211 (41.9)	145 (28.8)	147 (29.2)
Total	214,343 (84.2)	35,888 (14.1)	4336 (1.7)	254,567	144,151 (56.6)	98,181 (38.6)	12,235 (4.8)

Values are presented as n (%)

Gestational age categories are defined as 12–21 weeks, 22–27 weeks, and ≥28 weeks of gestation

Timing of death is classified as antepartum, intrapartum, or not specified (NS)

Condition at birth is classified as fresh (normal), macerated, or not specified (NS)

Percentages are calculated within each gestational age category

## Discussion

Applying ICD‑11 criteria to an eleven-year period of Mexican FD records, aligned with international reporting practices, yielded standardized late FD rates that are lower than reported national indicators. The recalculated late FD rate averaged 4.9 per 1,000 total births and increased to about 5.5 per 1,000 during 2020–2023.This pattern should be interpreted with caution, as it is strongly influenced by changes in the denominator, particularly the substantial decline in registered live births over the study period. These results indicate that much of the previously perceived fetal mortality burden in Mexico is derived from indicator definition (inclusion of all gestational ages and live‑birth‑only denominators) rather than from late‑gestation mortality per se [13, 16]. The increase in the rate occurs in parallel with a marked decline in total births, highlighting the influence of denominator dynamics.

The differences observed between official Mexican fetal death rates and the recalculated estimates are explained by several methodological factors. First, official statistics include fetal deaths from ≥ 12 weeks of gestation, whereas the present analysis applies ICD-11 criteria restricted to ≥ 28 weeks. Second, official rates are commonly calculated using live births as the denominator, while international standards define fetal death rates using total births (live births plus fetal deaths). Third, national statistics are derived from different data capture approaches, which may vary over time. Together, these differences limit the comparability of official estimates with international indicators. The use of standardized ICD-11 criteria in this study provides a more consistent framework for comparison across populations and overtime. These findings highlight the importance of aligning national reporting systems with international standards to ensure valid comparisons and support global monitoring of perinatal health.

Recalculated Mexican late FD rates are below the WHO/ENAP medium-term targets (≤ 12 by 2030 and ≤ 10 by 2035 per 1,000 total births) [1] and broadly consistent with the United Nations Inter-agency Group for Child Mortality Estimation (UN-IGME), with regional patterns showing declines in late FDs over the past two decades [4]. Nevertheless, the Mexican late FD rate remains nearly twice as high as the US level (2.7–2.9 per 1,000) [26, 28, 29], underscoring opportunities to strengthen intrapartum and late-gestation care.

The PAHO/WHO series places Mexico around 7–8 per 1,000 [25]; differences between PAHO/WHO estimates and the recalculated rates can be explained by variations in indicator construction. According to PAHO technical definitions, fetal mortality is calculated as the number of fetal deaths per 1,000 total births (live births plus fetal deaths), with fetal deaths restricted to those with a birthweight ≥ 1,000 g or, when birthweight is unavailable, to those occurring at ≥ 28 weeks of gestation. In contrast, the present study applies ICD-11 criteria directly to national microdata using a consistent definition based solely on gestational age (≥ 28 weeks) and a standardized denominator (total births), without combining criteria or applying additional adjustments. These differences in definition and data processing limit direct comparability and should be considered when interpreting cross-source estimates [13, 25].

In our work, nearly 60% of all FDs occurred before 28 weeks (very early and early), while in the US, early (22^+ 0^ to 27^+ 6^ weeks) FDs account for 52% and very early (12^+ 0^ to 21^+ 6^ weeks) FDs are not measured [26]. Because ICD-11 late FDs intentionally excludes < 28 weeks for international comparability [13], countries like Mexico should monitor both late and all gestational ages fetal mortality to inform comprehensive perinatal quality strategies. However, this finding should be interpreted with caution.

Early fetal deaths are particularly sensitive to changes in registration practices, clinical recognition, and administrative definitions, which may vary over time. In addition, the observed increase in the proportion of fetal deaths at earlier gestational ages and those with birthweight ≤ 499 g may reflect changes in reporting practices, improved detection, or classification differences rather than true epidemiological increases in early fetal loss. Furthermore, induced terminations of pregnancy cannot be identified in the available data, which may contribute to the observed patterns in very early gestational age and very low birthweight categories. These factors should be considered when interpreting trends in early fetal mortality. However, as with gestational age, these patterns should be interpreted cautiously given potential changes in registration and classification practices over time.

The apparent inconsistency between the high proportion of antepartum deaths and the relatively modest share of macerated skin suggests potential misclassification or recording issues. Possible explanations include variability in the assessment and recording of maceration, timing misclassification, and differential missingness across facilities. Similar discrepancies have been observed in multi-country low- and middle-income countries registries, where classification practices and time-to-delivery intervals influence the observed balance between “fresh” and “macerated” appearances [5, 7]. Strengthening certification practices (standardized training, clearer definitions, routine data quality audits) and, where feasible, introducing proportional redistribution procedures for “NS” gestational age—as implemented by the US National Center for Health Statistics (NCHS)—would improve comparability and reduce bias in trend analysis [26].

Our study has strengths and limitations. Strengths include the use of national administrative data with eleven-year of records, transparent application of ICD-11 definitions, and triangulation against national and international reference series. Limitations include: (i) lack of clinical causes of death; (ii) potential error in gestational age measurement; (iii) inconsistent reporting of skin condition and timing; and (iv) inability to identify induced terminations within the dataset [16, 19]. In Mexico, fetal deaths occurring at or beyond 12 weeks of gestation are expected to be registered in official statistics; however, induced abortions are not systematically distinguished in the available data. Therefore, the proportion of cases corresponding to induced terminations, particularly after 12 weeks, cannot be determined. The extent to which these events are consistently reported may vary depending on legal, administrative, and reporting practices. These legal considerations are complex and fall outside the scope of this study, but they may introduce heterogeneity in the classification of very early and early fetal deaths. However, it is unlikely to substantially affect the analysis of late fetal deaths (≥ 28 weeks), which are the primary focus of this study. And (v) use of different data sources for live births across the study period. While INEGI data were used for earlier years, more recent data were obtained from the Secretary of Health (DGIS), reflecting the increasing role of health information systems in national reporting. Both sources are part of the Mexican National System of Statistical and Geographic Information and are considered official data for national indicators. However, differences in data collection and registration processes may affect strict comparability and could have influenced the observed trends. Although the decline in live births was evident prior to this transition, this factor should be considered when interpreting temporal trends.

Three priorities emerge for policy and research. First, adopting dual reporting by publishing an ICD-11-aligned late FD rate (≥ 28 weeks per 1,000 total births) while also releasing an all-gestational age fetal mortality indicator for domestic program management and early-loss surveillance. Second, improving data quality through routine audits of FD certification, harmonized macros for plausibility checks (gestational age, birthweight, size), and explicit handling of “nonspecific” categories (e.g., proportional redistribution rules). Third, improving the interpretation and use of fetal death indicators requires strengthening measurement and surveillance systems. This includes enhancing data quality through standardized definitions, improved registration practices, and consistent handling of key variables such as gestational age and birthweight. Although differences in fetal death rates may suggest potential areas for improvement in perinatal care, the present study does not directly assess causes of death, clinical management, or quality-of-care indicators. Therefore, implications for healthcare quality and specific interventions should be interpreted with caution. Future research integrating clinical, facility-level, and cause-of-death data would be necessary to better understand the determinants of fetal mortality and identify actionable areas for improving care [1, 29].

Overall, the retrospective application of ICD-11 clarifies Mexico’s progress and its remaining gaps in fetal mortality. Standardized measurement enhances international comparability, supports accountability for perinatal care quality, and offers a more reliable baseline for evaluating future interventions and equity-focused strategies.

## Conclusions

Recalculating late FD rates in Mexico using ICD-11 criteria produced standardized and internationally comparable indicators that differ substantially from historically published national rates. Mexico is already below the WHO/ENAP thresholds, although it still maintains rates nearly twice as high as in the US, indicating areas for improvement in perinatal care. These findings highlight the need to improve the standardization of fetal death measurement in line with ICD-11 criteria, strengthen data quality and classification practices, and enhance perinatal care, particularly to prevent late fetal deaths (≥ 28 weeks).

## Data Availability

The datasets generated and/or analyzed during the current study are available in the [Estadísticas de Defunciones Fetales (EDF) , Datos abiertos] repository, [https://www.inegi.org.mx/programas/edf/#datos_abiertos] and the [Natalidad, Conjunto de datos: nacimientos , Datos abiertos] repository, [Natalidad, Conjunto de datos: nacimientos, Datos abiertos] repository, [https://www.inegi.org.mx/sistemas/olap/Proyectos/bd/continuas/natalidad/nacimientos.asp] All data generated or analyzed during this study are included in this published article.

## Abbreviations

CDC: Centers for Disease Control and Prevention
ENAP: Every Newborn Action Plan
FDs: Fetal deaths
ICD: International Classification of Diseases
ICD-10: International Classification of Diseases, 10th Revision
ICD-11: International Classification of Diseases, 11th Revision
INEGI: National Institute of Statistics and Geography
NCHS: National Center for Health Statistics
NS: Not specified
PAHO: Pan American Health Organization
SPSS: Statistical Package for the Social Sciences
STROBE: STROBE Statement for cross-sectional studies
UN-IGME: United Nations Inter-agency Group for Child Mortality Estimation
US: United States
WHO: World Health Organization
